# iComBat: An incremental framework for batch effect correction in DNA methylation array data

**DOI:** 10.1016/j.csbj.2025.09.014

**Published:** 2025-09-15

**Authors:** Yui Tomo, Ryo Nakaki

**Affiliations:** a National Institute of Infectious Diseases, Japan Institute for Health Security, 1-23-1 Toyama, Shinjuku-Ku, 162-0052, Tokyo, Japan; b Rhelixa, Inc., 3-7-2 Irifune, Chuo-ku, 104-0042, Tokyo, Japan

**Keywords:** ComBat, Empirical Bayes estimation, Epigenetics, Microarray, Repeated measurement

## Abstract

DNA methylation is associated with various diseases and aging; thus, longitudinal and repeated assessments of methylation patterns are crucial for revealing the mechanisms of disease onset and identifying factors associated with aging. The presence of batch effects influences the analysis of DNA methylation array data. As existing methods for correcting batch effects are designed to correct all samples simultaneously, when data are incrementally measured and included, the correction of newly added data affects previous data. Therefore, we propose an incremental framework for batch-effect correction based on ComBat, a location/scale adjustment approach using a Bayesian hierarchical model, and empirical Bayes estimation. Using numerical experiments and application to actual data, we demonstrate that the proposed method can correct newly included data without re-correcting the old data. The proposed method is expected to be useful for studies involving repeated measurements of DNA methylation, such as clinical trials of anti-aging interventions.

## Highlights

• We propose iComBat, an incremental batch effect correction method that allows newly added batches to be adjusted without reprocessing previously corrected data.
• As a modification of ComBat, a widely used batch correction method, iComBat inherits its strengths, such as robustness to small sample sizes within batches.
• Simulation studies and real-world data applications demonstrate the efficiency of iComBat.
• iComBat is particularly useful for longitudinal studies involving repeated measurements, such as clinical trials of anti-aging interventions based on DNA methylation or epigenetic clocks.

## Graphical Abstract

**Figure:**
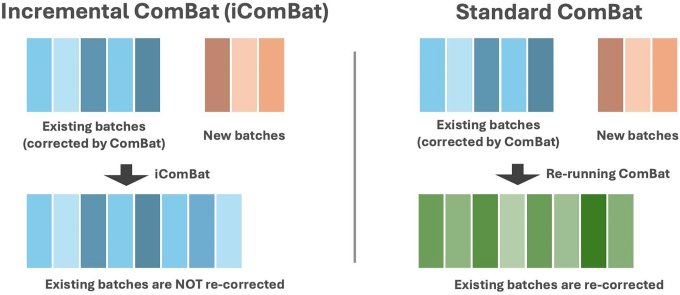

## 1 Introduction

DNA methylation is an epigenetic modification characterized by the addition of a methyl group to the cytosine base constituting a DNA molecule [1]. Although DNA methylation does not modify the DNA sequence itself, it plays a vital role in the regulation of gene expression and is related to a variety of biological processes. For example, DNA methylation is associated with the onset of various diseases, from cancer to infectious diseases [2], [3], [4]. Furthermore, studies have revealed a relationship between DNA methylation and aging [5], [6], [7]. Therefore, a comprehensive analysis of DNA methylation patterns is essential for revealing the mechanisms underlying disease pathogenesis and aging.

Recently, DNA methylation arrays have been widely used to analyze large-scale samples. Epigenome-wide association studies (EWAS) have assessed and identified associations between methylation at each measured methylation site and specific phenotypes [8]. In addition, formulas known as epigenetic clocks, which calculate the biological age from DNA methylation data, have been developed using statistical and machine learning approaches [9], [10], [11]. These epigenetic clocks are currently employed to evaluate anti-aging interventions and to assess the effects of aging-related exposures [12], [13]. However, batch effects present a major challenge in analyzing DNA methylation array data [14]. Batch effects are systematic variations arising from technical factors such as differences in instrumentation, reagent lots, measurement times, and other experimental conditions across batches. These effects may impede data analysis and potentially influence biological interpretations and clinical decision-making [15], [16]. Therefore, the correction of batch effects is critical for enhancing the reliability of data analyses.

Various statistical methods have been developed for batch-effect correction. Quantile normalization standardizes the distribution of signal intensities among samples under the assumption that signals of the same rank share the same intensity [17]. Surrogate variable analysis (SVA) and two-step removal of unwanted variation (RUV-2) methods adjust for unobserved sources of variability by extracting latent variables through a low-rank approximation of the residual matrix, which is obtained by regressing the signal intensity matrix on observed factors, and subsequently removing the associated variation through additional regression [18], [19]. ComBat is based on a location/scale (L/S) adjustment model that corrects data across batches by adjusting the mean and scale parameters of the observed data. In ComBat, the location and scale parameters for each gene are estimated using empirical Bayes methods within a hierarchical model that borrows information across genes in each batch [20]. ComBat has been widely adopted and extended in many studies as it works well even when sample sizes within batches are small [21], [22], [23].

In addition to post-hoc statistical corrections, preprocessing pipelines, such as SeSAMe, have been employed to reduce batch effects at the signal normalization stage. SeSAMe [24] is particularly effective in addressing technical biases stemming from array-specific features, including dye bias, background noise, and scanner variability. However, SeSAMe alone cannot fully correct all sources of batch effects. It remains limited in mitigating biological and experimental variations, such as differences in DNA extraction protocols, plate layouts, and bisulfite conversion efficiencies. These uncorrected sources of variation may affect the accuracy of the downstream DNA methylation Beta-value or M-value calculation. These findings highlight the need for complementary statistical correction methods.

Conventional methods for batch-effect correction have been designed to simultaneously correct data from all batches. However, in long-term studies, where data are repeatedly measured and evaluated, new batches are continuously added. In such scenarios, it is desirable to apply a consistent correction to the newly included data without modifying the already corrected existing data. In this study, we developed an incremental framework for batch-effect correction based on the L/S adjustment model and the Bayesian framework employed in ComBat. The proposed method is expected to facilitate the analysis of repeatedly included new batches without re-correcting previously corrected data and allow us to consistently interpret the results from the overall dataset.

The remainder of this paper is organized as follows: In Section 2, we describe the details of ComBat and propose an incremental batch effect correction framework based on ComBat. In Section 3, we describe the methods of numerical experiments and the application to actual datasets to evaluate the performance of our proposed method. In Section 4, we present the results. In Section 5, we discuss the strengths of our proposed method and scope for future research.

## 2 Theory/calculation

### 2.1 Review of batch effect correction using ComBat

ComBat employs a statistical model that accounts for additive and multiplicative batch effects to eliminate these effects [20]. Furthermore, the model was designed as a Bayesian hierarchical model to borrow information across methylation sites within each batch, which is expected to perform stably even with small sample sizes. This estimation is based on an empirical Bayes approach [25].

To formulate the model and estimation method, we introduce the following notation: Let $Y_{ijg}\in R$ denote the M-value for batch i, sample j, and methylation site g (i=1,…,m, $j=1,\ldots ,n_{i}$, and g=1,…,G). The M-value is defined as the log-ratio of the intensities of the methylated and unmethylated signals:

$$\begin{array}{l} Y_{ijg}:=\log_{2}\left(\frac{M_{ijg}+\eta }{U_{ijg}+\eta }\right), \end{array}$$

where $M_{ijg}>0$ denotes methylated signal intensities and $U_{ijg}>0$ denotes unmethylated [26]. Here, η>0 is a small positive constant added to numerical stability. The M-value can be approximately converted to the beta-value, defined as

$$\begin{array}{l} B_{ijg}:=\frac{M_{ijg}}{M_{ijg}+U_{ijg}+\eta }, \end{array}$$

approximately obtained from M-value via the following transformation:

$$\begin{array}{l} B_{ijg}≃\frac{2^{Y_{ijg}}}{2^{Y_{ijg}}+1}. \end{array}$$

Let $X_{ij}\in R^{p}$ denote the covariate vector representing the sample conditions and let $\beta_{g}\in R^{p}$ denote the corresponding regression coefficients. Furthermore, let $\alpha_{g}\in R$ denote the site-specific effect for methylation site g and let $\gamma_{ig}\in R$ and $\delta_{ig}\in R$ denote the additive and multiplicative effects for batch i, respectively. ComBat then assumes the following model:

$$\begin{array}{l} Y_{ijg}=\alpha_{g}+X_{ij}^{⊤}\beta_{g}+\gamma_{ig}+\delta_{ig}\varepsilon_{ijg}, \end{array}$$

where $\varepsilon_{ijg}∼N(0,\sigma_{g}^{2})$ denotes the error term with standard deviation $\sigma_{g}>0$. The model estimation and batch effect correction are performed in the following three steps:

#### Step 1: Estimation of the global parameters

Global parameters $\alpha_{g}$, $\beta_{g}$, and $\sigma_{g}$ for each methylation site g are estimated to standardize the observed data so that the mean and variance are equal across methylation sites. First, we consider a model that includes only the additive batch effect.

$$\begin{array}{l} Y_{ijg}=\alpha_{g}+X_{ij}^{⊤}\beta_{g}+\gamma_{ig}+\varepsilon_{ijg}. \end{array}$$

Let $Y_{g}\in R^{N_{m}}$ denote a vector of observations, defined as follows:

$$\begin{array}{l} Y_{g}:={\left(Y_{11g},\ldots ,Y_{1n_{1}g},\;Y_{21g},\ldots ,Y_{2n_{2}g},\;\ldots ,\;Y_{m1g},\ldots ,Y_{mn_{m}g}\right)}^{⊤}, \end{array}$$

where $N_{m}=\sum_{i=1}^{m}n_{i}$ denotes the total number of samples. Let $X\in R^{N_{m}\times (m+p)}$ be the design matrix constructed from the batch indicator variables and other covariates, i.e., $X:=(X_{\text{batch}},\;X_{\text{cov}})$, where $X_{\text{batch}}\in R^{N_{m}\times m}$ is the matrix of indicator variables for the m batches constructed without a constant column and $X_{\text{cov}}\in R^{N_{m}\times p}$ is the matrix of the p covariates defined as

$$\begin{array}{l} X_{\text{cov}}={\left(X_{11},\ldots ,X_{1n_{1}},\;X_{21},\ldots ,X_{2n_{2}},\;\ldots ,\;X_{m1},\ldots ,X_{mn_{m}}\right)}^{⊤}. \end{array}$$

The estimates ${\hat{\alpha }}_{g}$, ${\hat{\beta }}_{g}$, and ${\hat{\sigma }}_{g}$ are then calculated under the identifiability condition $\sum_{i=1}^{m}n_{i}\gamma_{ig}=0$ for g=1,…,G. First, the ordinary least squares solution ${\hat{\vartheta }}_{g}\in R^{\left(m+p\right)}$ is calculated as

$$\begin{array}{l} {\hat{\vartheta }}_{g}={\left(X^{⊤}X\right)}^{-1}X^{⊤}Y_{g}, \end{array}$$

where ${\hat{\vartheta }}_{g}$ can be partitioned as:

$$\begin{array}{l} {\hat{\vartheta }}_{g}={\left({\hat{\gamma }}_{1g}^{\prime },\;{\hat{\gamma }}_{2g}^{\prime },\;\ldots ,\;{\hat{\gamma }}_{mg}^{\prime },\;{\hat{\beta }}_{g}^{⊤}\right)}^{⊤}, \end{array}$$

where ${\hat{\gamma }}_{ig}^{\prime }$ is the estimate of $\gamma_{ig}$ without centralization. Then, ${\hat{\alpha }}_{g}$ is calculated as:

$$\begin{array}{l} {\hat{\alpha }}_{g}=\frac{1}{N_{m}}\sum_{i=1}^{m}n_{i}\;{\hat{\gamma }}_{ig}^{\prime }, \end{array}$$

and ${\hat{\gamma }}_{ig}$ is ${\hat{\gamma }}_{ig}={\hat{\gamma }}_{ig}^{\prime }-{\hat{\alpha }}_{g}$ satisfying $\sum_{i=1}^{m}n_{i}{\hat{\gamma }}_{ig}=0$. Furthermore, the variance $\sigma_{g}^{2}$ is estimated as:

$$\begin{array}{l} {\hat{\sigma }}_{g}^{2}=\frac{1}{N_{m}}\sum_{ij}{\left(Y_{ijg}-{\hat{\alpha }}_{g}-X_{ij}^{⊤}{\hat{\beta }}_{g}-{\hat{\gamma }}_{ig}\right)}^{2}. \end{array}$$

#### Step 2: Estimation of the batch effect parameters

Using the estimates of global parameters, the observed data are standardized as follows:

$$\begin{array}{l} Z_{ijg}=\frac{Y_{ijg}-{\hat{\alpha }}_{g}-X_{ij}^{⊤}{\hat{\beta }}_{g}}{{\hat{\sigma }}_{g}}. \end{array}$$

We assume the following Bayesian hierarchical model for the standardized data $Z_{ijg}$:

$$\begin{array}{ll} Z_{ijg} & \;∼N(\gamma_{ig},\delta_{ig}^{2}),\\ \gamma_{ig} & \;∼N(\gamma_{i},\tau_{i}^{2}),\\ \delta_{ig}^{2} & \;∼\text{InverseGamma}(\zeta_{i},\theta_{i}). \end{array}$$

The hyperparameter estimates ${\bar{\gamma }}_{i}$, ${\bar{\tau }}_{i}^{2}$, ${\bar{\zeta }}_{i}$, and ${\bar{\theta }}_{i}$ are computed using the method of moments, and are obtained as

$$\begin{array}{ll} {\bar{\gamma }}_{i} & \;=\frac{1}{G}\sum_{g=1}^{G}{\hat{\gamma }}_{ig},\\ {\bar{\tau }}_{i}^{2} & \;=\frac{1}{G-1}\sum_{g=1}^{G}{\left({\hat{\gamma }}_{ig}-{\bar{\gamma }}_{i}\right)}^{2},\\ {\bar{\zeta }}_{i} & \;=\frac{a^{2}}{s^{2}}+2,\\ {\bar{\theta }}_{i} & \;=a\left(\frac{a^{2}}{s^{2}}+1\right), \end{array}$$

where $a=\sum_{g=1}^{G}{\hat{\delta }}_{ig}^{2}/G$, $s^{2}=\sum_{g=1}^{G}{\left({\hat{\delta }}_{ig}^{2}-a\right)}^{2}/(G-1)$, and ${\hat{\delta }}_{ig}{}^{2}=\sum_{j}{\left(Z_{ijg}-{\hat{\gamma }}_{ig}\right)}^{2}/(n_{i}-1)$. Then, the empirical Bayes estimators ${\hat{\gamma }}_{ig}^{*}$ and ${\hat{\delta }}_{ig}^{2*}$ of batch effects satisfy the following simultaneous equations:

$$\begin{array}{ll} {\hat{\gamma }}_{ig}^{*} & \;=\frac{n_{i}{\bar{\tau }}_{i}^{2}\;{\hat{\gamma }}_{ig}+{\hat{\delta }}_{ig}^{2*}\;{\bar{\gamma }}_{i}}{n_{i}{\bar{\tau }}_{i}^{2}+{\hat{\delta }}_{ig}^{2*}},\;{\hat{\delta }}_{ig}^{2*}=\frac{{\bar{\theta }}_{i}+\sum_{j}{\left(Z_{ijg}-{\hat{\gamma }}_{ig}^{*}\right)}^{2}/2}{n_{i}/2+{\bar{\lambda }}_{i}-1}, \end{array}$$

and are obtained as numerical solutions through iterative updates.

#### Step 3: Data correction

Finally, using the estimated global and batch effect parameters, the corrected data are obtained as follows:

$$\begin{array}{l} Y_{ijg}^{*}=\frac{{\hat{\sigma }}_{g}}{{\hat{\delta }}_{ig}^{*}}\left(Z_{ijg}-{\hat{\gamma }}_{ig}^{*}\right)+{\hat{\alpha }}_{g}+X_{ij}^{⊤}{\hat{\beta }}_{g}. \end{array}$$

### 2.2 Proposed incremental framework: incremental ComBat (iComBat)

We propose Incremental ComBat (iComBat), an incremental framework based on ComBat that corrects a newly included batch, avoiding modifications to the existing correction results. This section describes the proposed method in detail.

After applying ComBat to the existing batches i=1,…,m, the estimated parameters for the correction model for each existing batch and for each site g=1,…,G, namely ${\hat{\alpha }}_{g}$, ${\hat{\beta }}_{g}$, ${\hat{\sigma }}_{g}$, ${\hat{\gamma }}_{ig}^{*}$, ${\hat{\delta }}_{ig}^{*}$, and the hyperparameter values of the prior distributions ${\bar{\gamma }}_{i}$, ${\bar{\tau }}_{i}^{2}$, ${\bar{\zeta }}_{i}$, and ${\bar{\theta }}_{i}$, are obtained. For a newly added batch i=m+1, we propose correcting the new data by estimating the batch effect parameters ${\hat{\gamma }}_{(m+1)g}^{*}$ and ${\hat{\delta }}_{(m+1)g}^{2*}$ using the new batch and the global parameters estimated using the existing batches {1,…,m}. Specifically, to standardize the methylation values $Y_{(m+1)jg}$ in the new batch m+1 at each site g, the estimated parameters ${\hat{\alpha }}_{g}$, ${\hat{\beta }}_{g}$, and ${\hat{\sigma }}_{g}$ from existing batches are employed. That is,

$$\begin{array}{l} Z_{(m+1)jg}=\frac{Y_{(m+1)jg}-{\hat{\alpha }}_{g}-X_{(m+1)j}^{⊤}{\hat{\beta }}_{g}}{{\hat{\sigma }}_{g}}, \end{array}$$

Using the standardized data $Z_{(m+1)jg}$, the estimates ${\hat{\gamma }}_{(m+1)g}^{*}$ and ${\hat{\delta }}_{(m+1)g}^{2*}$ are obtained using the same procedure as in Step 2 of the conventional ComBat. The corrected data for the new batch are then computed as:

$$\begin{array}{l} Y_{(m+1)jg}^{*}=\frac{{\hat{\sigma }}_{g}}{{\hat{\delta }}_{(m+1)g}^{*}}\left(Z_{(m+1)jg}-{\hat{\gamma }}_{(m+1)g}^{*}\right)+{\hat{\alpha }}_{g}+X_{(m+1)j}^{⊤}{\hat{\beta }}_{g}. \end{array}$$

Subsequently, the corrected values $Y_{(m+1)jg}^{*}$ are obtained on the same scale as those of the existing batches.

## 3 Materials and methods

### 3.1 Numerical experiments

We evaluated the performance of the proposed method using numerical experiments. For methylation sites g∈{1,…,G} and samples $j\in \{1,\ldots ,n_{i}\}$, data $Y_{ijg}$ belonging to batch i∈{1,…,m} were generated according to the following model:

$$\begin{array}{l} Y_{ijg}=\left(\mu_{\text{global}}+\varepsilon_{ijg}+\nu_{i}+X_{ij}\Delta_{g}+A_{ij}\beta_{g}\right)\cdot \sigma_{i}, \end{array}$$

where $\mu_{\text{global}}$ is the global mean, $\varepsilon_{ijg}∼N(0,\sigma_{\text{global}}^{2})$ is the noise term with global variance $\sigma_{\text{global}}^{2}$, $\nu_{i}$ is the mean shift owing to batch i, and $\sigma_{i}$ is the scaling factor for batch i. $X_{ij}\in [0,1]$ is an indicator variable representing the group to which sample j belongs (e.g., 0: the control group; 1: the treatment group). $\Delta_{g}$ denotes the group effect, $A_{ij}$ represents the age of sample j in batch i, and $\beta_{g}$ denotes the age effect for site g.

For the data generation, the total number of sites was set to G=500. To introduce differential methylation between the control and treatment groups only for the first 50 sites, we set $\Delta_{g}=0.5$ for g=1,…,50, and $\Delta_{g}=0$ for g=51,…,500. Age effects were set to $\beta_{g}=0.01$ for g=1,…,500. Ages were sampled from a normal distribution $N(50,10^{2})$. The global parameters were set to $\mu_{\text{global}}=5$ and $\sigma_{\text{global}}=1$. We first set the parameters of the baseline scenario as follows. For the existing batches, we used m=3 and set the following parameters: for batch 1, $\left(n_{1},\nu_{1},\sigma_{1},p_{1})=(50,0,1,0.5\right)$; for batch 2, $\left(n_{2},\nu_{2},\sigma_{2},p_{2})=(30,2.5,1.5,0.5\right)$; and for batch 3, $\left(n_{3},\nu_{3},\sigma_{3},p_{3})=(40,-2,1.2,0.5\right)$, where $p_{i}$ denotes the probability of assigning a sample to the treatment group in batch i. Thus, the data from the existing batches comprised 120 samples. Furthermore, a new batch (m+1=4) was added with parameters $\left(n_{4},\nu_{4},\sigma_{4},p_{4})=(30,-4,1.8,0.5\right)$.

In addition to this baseline scenario, we evaluated the performance across 12 additional scenarios. These scenarios included variations in sample sizes, number of batches, case-control imbalance, age effect strength, and combinations of these. The detailed descriptions of all 13 scenarios are provided in Table 1, and the specific batch parameters $\left(n_{i},\nu_{i},\sigma_{i},p_{i}\right)$ for each scenario are presented in Table 2.

**Table 1:** Sample sizes, number of batches, and age effect for simulation scenarios.

Scenario	Description	Total	Batches	Age effect
		samples	(existing + new)	($\beta_{g}$)
Basic scenario				
S1	Baseline	150	3+1	0.01
Case-control imbalance scenarios				
S2	Mild imbalance	150	3+1	0.01
S3	Extreme imbalance	150	3+1	0.01
Sample size variations				
S4	Small samples	75	3+1	0.01
S5	Large samples	750	3+1	0.01
S6	Mixed sizes	360	3+1	0.01
Batch number variations				
S7	Few batches	120	2+1	0.01
S8	Many batches	165	6+3	0.01
Strong covariate effect				
S9	Strong covariate	150	3+1	0.10
S10	Extremely strong covariate	150	3+1	1.00
Complex scenarios				
S11	Imbalance + strong covariate	150	3+1	0.10
S12	Imbalance + strong covariate	75	3+1	0.10
	+ small samples			
S13	Imbalance + strong covariate	60	2+1	0.10
	+ small samples + few batches

**Table 2:** Batch parameters for each simulation scenario. All scenarios use G=500 sites, $\mu_{\text{global}}=5$, $\sigma_{\text{global}}=1$, $\Delta_{g}=0.5$ for sites 1–50 and $\Delta_{g}=0$ for sites 51–500, $\mu_{\text{age}}=50$, and $\sigma_{\text{age}}=10$.

Scenario	Batch Type	$\left(n_{i},\nu_{i},\sigma_{i},p_{i}\right)$ for each batch		
S1	Existing	(50, 0, 1.0, 0.5)	(30, 2.5, 1.5, 0.5)	(40, −2, 1.2, 0.5)
	New	(30, −4, 1.8, 0.5)		
S2	Existing	(50, 0, 1.0, 0.3)	(30, 2.5, 1.5, 0.7)	(40, −2, 1.2, 0.4)
	New	(30, −4, 1.8, 0.6)		
S3	Existing	(50, 0, 1.0, 0.1)	(30, 2.5, 1.5, 0.9)	(40, −2, 1.2, 0.2)
	New	(30, −4, 1.8, 0.8)		
S4	Existing	(25, 0, 1.0, 0.5)	(15, 2.5, 1.5, 0.5)	(20, −2, 1.2, 0.5)
	New	(15, −4, 1.8, 0.5)		
S5	Existing	(250, 0, 1.0, 0.5)	(150, 2.5, 1.5, 0.5)	(200, −2, 1.2, 0.5)
	New	(150, −4, 1.8, 0.5)		
S6	Existing	(200, 0, 1.0, 0.5)	(10, 2.5, 1.5, 0.5)	(50, −2, 1.2, 0.5)
	New	(100, −4, 1.8, 0.5)		
S7	Existing	(50, 0, 1.0, 0.5)	(30, 2.5, 1.5, 0.5)	
	New	(40, −2, 1.2, 0.5)		
S8	Existing	(20, 0, 1.0, 0.5)	(20, 0.5, 1.1, 0.5)	(20, 1, 1.2, 0.5)
		(20, −0.5, 1.1, 0.5)	(20, −1, 1.2, 0.5)	(20, 1.5, 1.3, 0.5)
	New	(15, −2, 1.4, 0.5)	(15, 2.5, 1.5, 0.5)	(15, −3, 1.6, 0.5)
S9	Existing	(50, 0, 1.0, 0.5)	(30, 2.5, 1.5, 0.5)	(40, −2, 1.2, 0.5)
	New	(30, −4, 1.8, 0.5)		
S10	Existing	(50, 0, 1.0, 0.5)	(30, 2.5, 1.5, 0.5)	(40, −2, 1.2, 0.5)
	New	(30, −4, 1.8, 0.5)		
S11	Existing	(50, 0, 1.0, 0.3)	(30, 2.5, 1.5, 0.7)	(40, −2, 1.2, 0.4)
	New	(30, −4, 1.8, 0.6)		
S12	Existing	(25, 0, 1.0, 0.3)	(15, 2.5, 1.5, 0.7)	(20, −2, 1.2, 0.4)
	New	(15, −4, 1.8, 0.6)		
S13	Existing	(25, 0, 1.0, 0.3)	(15, 2.5, 1.5, 0.7)	
	New	(20, −2, 1.2, 0.4)		

iComBat was then applied to the generated data. The first three batches were corrected using the conventional ComBat, and the new batch was corrected using the global parameters obtained from the already applied ComBat. For comparison, simultaneous batch correction was performed for all four batches using ComBat. The experiment was repeated 1000 times. For uncorrected data, data corrected using ComBat, and data corrected using iComBat, linear regression analysis was performed for each methylation site to compare the treatment and control groups while adjusting for age as a covariate. Using a significance level of 0.05, the true-positive rate (TPR) and false-positive rate (FPR) were calculated. Additionally, we computed the genomic control inflation factor (GC λ) and the number of surrogate variables (nSV) detected by SVA. In addition, principal component analysis (PCA) was applied to the combined data from the existing and new batches, and scatter plots were drawn using the first principal component (PC1), the second (PC2), and the third (PC3).

### 3.2 Application to actual data

To demonstrate the practical performance of iComBat, we employed three publicly available DNA methylation datasets [27], [28], [29]. The first two datasets were used for epigenome-wide association studies (EWAS), while the third was used for epigenetic clock evaluation.

#### Dataset 1: GSE42861 – rheumatoid arthritis and smoking EWAS

We employed the publicly available GSE42861 dataset, which contains Illumina HumanMethylation450 measurements from whole-blood DNA of healthy controls and patients with rheumatoid arthritis [27]. We used the authors’ preprocessed beta-value dataset, with the quality control details described in the original publication. After excluding probes on X/Y chromosomes and probes with zero variance, G=473,925 CpG sites were retained for analysis. The dataset comprised 689 samples with clinical annotations for rheumatoid arthritis status and smoking history.

We conducted two separate EWAS analyses. In the first analysis, we examined the association with rheumatoid arthritis by comparing 354 cases to 335 healthy controls. In the second analysis, we investigated smoking-related methylation sites by comparing 428 individuals with a history of smoking to 261 who had never smoked. For both analyses, we adjusted for age, sex, rheumatoid arthritis, and smoking history as covariates. When rheumatoid arthritis was the outcome, smoking history was included as a covariate; when smoking history was the outcome, rheumatoid arthritis status was included as a covariate. Beta-values were transformed to M-values for all analyses. Differential methylation was assessed using linear models with empirical Bayes moderation [30], implemented using the lmFit and eBayes functions from the limma package [31] .

The 72 Sentrix array identifiers were treated as batches. For the iComBat evaluation, we designated 40 arrays (identifiers beginning with “5") as existing batches and 32 arrays (identifiers beginning with “7") as new batches to be incrementally integrated. Both ComBat and iComBat batch corrections were performed with adjustments for age, sex, rheumatoid arthritis status, and smoking history as biological covariates.

We compared the following three scenarios: (i) no correction: raw M-values from 72 batches; (ii) corrected using ComBat: all 72 batches corrected using the standard ComBat simultaneously; (iii) corrected using iComBat: the first 40 batches were designated as the existing batches and corrected using ComBat; the remaining 32 batches were then incrementally corrected using iComBat and global parameter values from the initial ComBat. Results were evaluated using PCA, sample-to-sample density plots between ComBat and iComBat, detected differential methylation, GC λ, and nSV. Furthermore, associations between the first three principal components and covariates (age, sex, batch) were tested using correlation analysis, t-tests, and analysis of variance (ANOVA), respectively. PCA was applied using the top 10,000 most variable CpG sites. Sample-to-sample density plots were constructed using a random sample of 100,000 CpG sites. If there were missing values in the M-values, they were imputed using the median of each site during evaluation. The significance level for EWAS was set at 0.05 and corrected using the Bonferroni method: $0.05/473,925≃1.06\times 10^{-7}$.

#### Dataset 2: GSE224218 – intracranial ependymoma EWAS

The GSE224218 dataset contains DNA methylation profiles from intracranial ependymoma tumor samples measured on both Illumina HumanMethylation450 (450 K, n=22) and EPIC (n=148) platforms [28]. Raw IDAT files were processed using the SeSAMe [24] with the following quality control steps: detection p-values were calculated using the pOOBAH (p-values by out-of-band array hybridization) method with a threshold of 0.01, normalization was performed using Noob (normal-exponential out-of-band) background correction followed by dye-bias correction, and probes failing detection in more than 10 % of samples were excluded. After identifying common probes between the two platforms and removing sex chromosome probes, G=167,085 CpG sites were retained for analysis. Among the 170 total samples, 163 (450 K: n=21, EPIC: n=142) had complete covariate information and were included in the analyses.

We performed three EWAS analyses. In the first analysis, we compared 65 patients with disease progression to 98 without progression. In the second analysis, we compared 37 patients who died to 126 survivors. In the third analysis, we examined tumor location by comparing 110 infratentorial to 53 supratentorial tumors. In all the analyses, we adjusted for age and sex as covariates. As with Dataset 1, beta-values were transformed to M-values and differential methylation was assessed using linear models with empirical Bayes moderation.

We used the measurement platform (450 K versus EPIC) as the batch variable. We evaluated iComBat in both directions: first designating EPIC as the existing batch and incrementally adding 450 K samples, then reversing the order with 450 K as the existing batch and EPIC as new. In both ComBat and iComBat corrections, age, sex, and three outcome variables used in EWAS analyses were adjusted as biological covariates. The four correction scenarios (raw, ComBat, iComBat: EPIC to 450 K, and iComBat: 450 K to EPIC) were compared using the same evaluation methods and metrics as Dataset 1. The Bonferroni-corrected significance level for EWAS was $0.05/167,085≃2.99\times 10^{-7}$.

#### Dataset 3: GSE286313 – evaluation of epigenetic age

The GSE286313 dataset comprises DNA methylation profiles from four separate cohorts measured on both EPICv1 and EPICv2 platforms [29]. For this analysis, we focused exclusively on EPICv2 samples. The dataset includes samples from CALERIE (United States of America, n=24), BeCOME (Germany, n=8), CLHNS (Philippines, n=16), and VHAS (Vietnam, n=24), for a total of 72 samples.

Raw IDAT files were processed using the SeSAMe pipeline with quality control parameters identical to Dataset 2. After removing sex chromosome probes, G=837,855 CpG sites were retained for analysis.

We calculated epigenetic age using the Horvath clock [9] to evaluate the stability of age predictions under different batch correction scenarios. Two approaches were compared. First, cohorts were added sequentially with ComBat applied at each step: CALERIE alone (uncorrected), CALERIE+BeCOME (corrected by ComBat), CALERIE+BeCOME + CLHNS (corrected by ComBat), and finally all four cohorts (corrected by ComBat). Second, we applied iComBat incrementally: CALERIE served as the existing batch (uncorrected), then BeCOME, CLHNS, and VHAS were added and corrected incrementally as new batches using iComBat.

Both ComBat and iComBat corrections were conducted with adjustments for chronological age and sex as biological covariates. Since variance estimation was unstable due to a small sample size, only the mean was corrected by ComBat and iComBat. The evaluation metric was the change in epigenetic age for samples in existing batches at each incremental step as new batches were added. Furthermore, we corrected all four cohorts simultaneously using ComBat (All-Batch ComBat) and compared it with the incremental scenario using iComBat. Other evaluation methods and metrics were the same as Dataset 1, except for EWAS-related metrics.

## 4 Results

### 4.1 Numerical experiments

Table 3, Table 4 present the comprehensive evaluation results across all 13 simulation scenarios. The baseline scenario (S1) revealed that while uncorrected data yielded an average TPR of 0.205, ComBat and iComBat achieved average TPRs of 0.827 and 0.877, respectively. The average FPR was 0.053 in uncorrected raw data and decreased to 0.040 for ComBat while slightly increasing to 0.067 for iComBat. The average GC λ was effectively reduced from 2.011 in uncorrected data to 1.173 for ComBat and 1.465 for iComBat. The average nSV was also reduced from 1.477 in uncorrected data to 0.248 for ComBat and 0.165 for iComBat.

**Table 3:** Average true positive rate (TPR) and false positive rate (FPR) comparison of Raw, ComBat, and iComBat across 13 simulation scenarios. Values shown are averages from 20 simulation runs.

Scenario	TPR			FPR		
	Raw	ComBat	iComBat	Raw	ComBat	iComBat
S1: Baseline	0.205	0.827	0.877	0.053	0.040	0.067
S2: Mild imbalance	0.853	0.844	0.876	0.422	0.048	0.077
S3: Extreme imbalance	1.000	0.828	0.822	0.972	0.113	0.165
S4: Small samples	0.122	0.538	0.615	0.053	0.048	0.076
S5: Large samples	0.705	1.000	1.000	0.051	0.036	0.060
S6: Mixed sizes	0.810	0.995	0.999	0.049	0.031	0.080
S7: Few batches	0.153	0.753	0.757	0.048	0.048	0.051
S8: Many batches	0.318	0.892	0.916	0.054	0.058	0.081
S9: Strong covariate	0.178	0.812	0.868	0.055	0.041	0.067
S10: Extremely strong covariate	0.040	0.176	0.382	0.038	0.017	0.047
S11: Imbalance + strong covariate	0.981	0.820	0.863	0.834	0.052	0.081
S12: Imbalance + strong covariate	0.796	0.543	0.609	0.536	0.060	0.092
+ small samples						
S13: Imbalance + strong covariate	0.813	0.467	0.476	0.642	0.067	0.081
+ small samples + few batches

**Table 4:** Average genomic control inflation factor (GC λ) and number of surrogate variables (nSV) across 13 simulation scenarios. Values shown are averages from 20 simulation runs.

Scenario	GC λ			nSV		
	Raw	ComBat	iComBat	Raw	ComBat	iComBat
S1: Baseline	2.011	1.173	1.465	1.477	0.248	0.165
S2: Mild imbalance	9.214	1.263	1.585	1.701	0.151	0.190
S3: Extreme imbalance	28.278	1.958	2.542	1.903	0.931	0.010
S4: Small samples	1.948	1.208	1.511	1.214	0.524	0.180
S5: Large samples	2.045	1.120	1.400	2.248	0.000	0.000
S6: Mixed sizes	1.742	1.068	1.612	1.563	0.000	0.000
S7: Few batches	2.001	1.242	1.276	1.933	0.398	0.217
S8: Many batches	1.843	1.371	1.624	2.800	1.000	0.562
S9: Strong covariate	2.041	1.185	1.479	1.605	0.732	0.876
S10: Extremely strong covariate	2.095	1.113	1.578	1.632	2.033	1.923
S11: Imbalance + strong covariate	21.083	1.305	1.622	1.721	0.734	0.974
S12: Imbalance + strong covariate	11.500	1.335	1.663	1.167	0.635	0.645
+ small samples						
S13: Imbalance + strong covariate	14.080	1.376	1.496	1.418	0.769	0.157
+ small samples + few batches						

Across different scenarios, the performance of iComBat was similar to ComBat in terms of all evaluation metrics. The correlation between ComBat and iComBat results remained extremely high across all scenarios (0.981–0.999). Notably, the presence of strong age effects (S9–S13) substantially impacted the performance of both methods. In scenario S10 with extremely strong covariate effects, both methods exhibited reduced performance with average TPRs of 0.176 (ComBat) and 0.382 (iComBat).

**Fig. 1:**
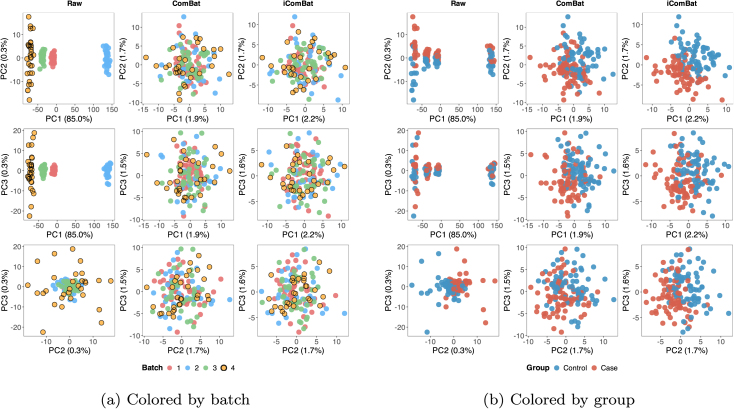
PCA plots of simulated data comprising existing batches 1–3 and a newly added batch 4. Each row shows different principal component combinations (PC1 vs PC2, PC1 vs PC3, PC2 vs PC3), and each column represents different correction methods: Raw (uncorrected), ComBat (all batches corrected together), and iComBat (batches 1–3 pre-corrected, batch 4 aligned incrementally). (a) Plots colored by batch membership, with new batch 4 highlighted with black borders. (b) Same data colored by group membership (Control vs Case).

Fig. 1 shows the PCA plots of the uncorrected data and the data corrected using each method for the baseline scenario (S1). For uncorrected data, the data from each batch were separated on PC1. However, for the data corrected using either ComBat or iComBat, a new batch (batch 4) was mapped such that it overlapped with existing batches (batches 1, 2, and 3). Thus, iComBat performs batch correction for new data without altering the correction results of the existing batches, while preserving the systematic signal associated with the assigned treatment.

### 4.2 Application to actual data

#### Dataset 1: GSE42861 – rheumatoid arthritis and smoking EWAS

**Fig. 2:**
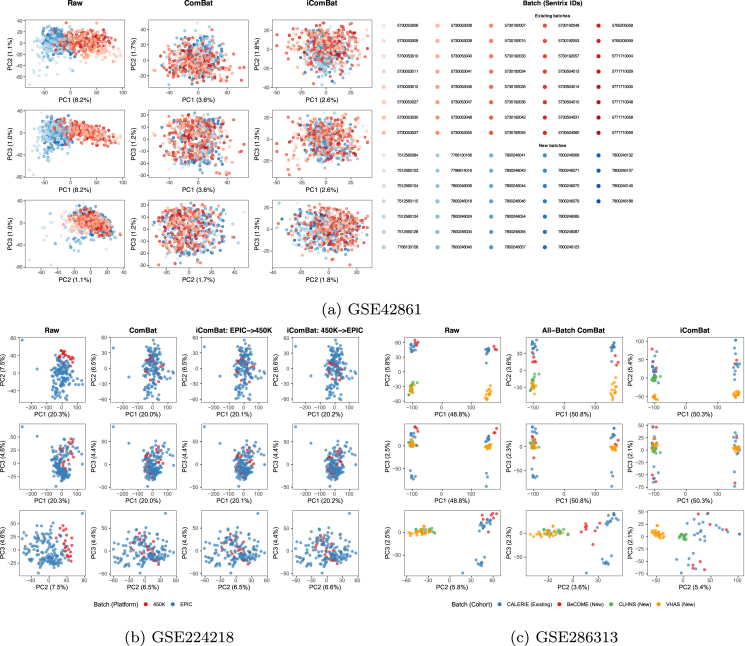
PCA plots of raw and corrected M-value from three datasets: (a) GSE42861, (b) GSE224218, and (c) GSE286313. For each subplot, the column-wise plots represent correction scenarios: Raw uncorrected data (column “Raw”), data corrected using standard ComBat (column “ComBat” or “All-Batch ComBat”), and data including additional batches processed with iComBat aligned with ComBat-corrected batches (column “iComBat”, “iComBat (EPIC → 450 K)” or “iComBat (450 K → EPIC)”).

**Fig. 3:**
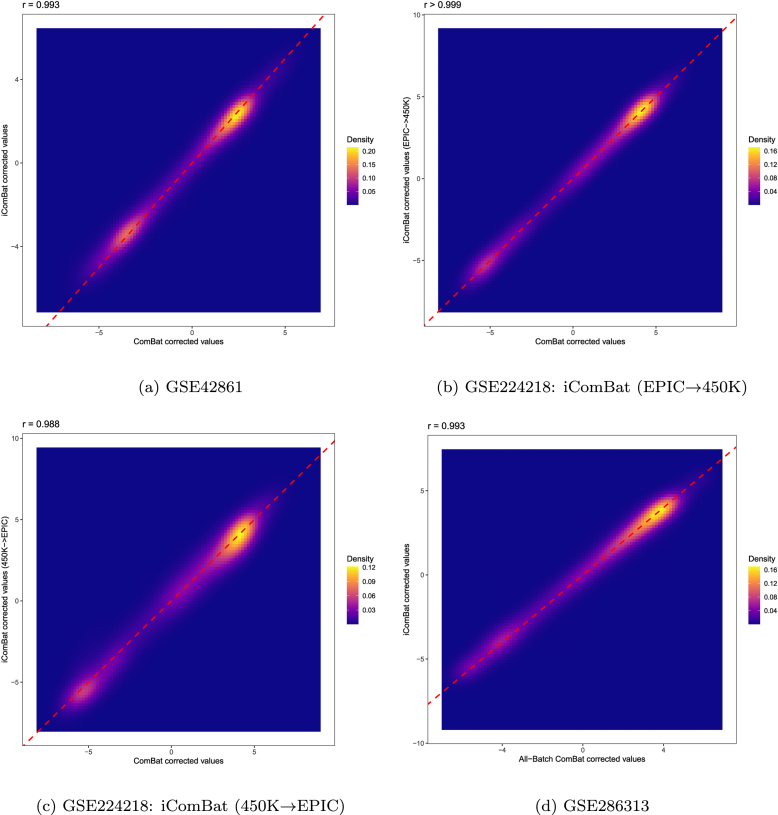
Sample-to-sample density plots of corrected values by ComBat and iComBat from three datasets: (a) GSE42861, (b, c) GSE224218, and (d) GSE286313.

Fig. 2a shows scatter plots using PC1 to PC3 for each correction scenario. In the raw, uncorrected data, distinct batch clusters were observed along PC1. After applying either ComBat or iComBat correction, these clusters overlapped. Fig. 3a presents the sample-to-sample density plot between ComBat and iComBat corrected data, showing a correlation of 0.993. Table 5 shows the GC λ for each EWAS analysis and nSV. While nSV remained increased in iComBat (4 for raw, 6 for ComBat, and 18 for iComBat), GC λ for the rheumatoid arthritis EWAS notably reduced, from 22.7 in raw data to 12.7 with ComBat and further to 3.62 with iComBat. Table 6 demonstrates that the association of batch with PCs in raw data (p<0.001 for all PCs) was effectively removed after ComBat and iComBat correction, while biological associations with age and sex were preserved.

**Table 5:** Genomic control inflation factor (GC λ) for each EWAS outcome and number of surrogate variables (nSV) across datasets and batch correction methods.

Dataset	Metric	Raw	ComBat	iComBat	
Dataset 1	GC λ				
(GSE42861)	Rheumatoid arthritis	22.7	12.7	5.66	
	Smoking history	1.12	1.24	1.34	
	nSV	4	6	18	
Dataset 2	GC λ			EPIC → 450 K	450 K → EPIC
(GSE224218)	Progression	1.21	1.34	1.30	1.30
	Death	1.80	1.79	1.73	1.76
	Tumor location	4.04	4.27	4.11	4.31
	nSV	153	156	156	156
Dataset 3	nSV	64	1	2	
(GSE286313)

**Table 6:** Association analysis between principal components and variables across three datasets. Statistics for variables are as follows: Age: Pearson correlation coefficient; Sex:t-statistic; Batch: ANOVA F-statistic (GSE42861, GSE286313) or t-statistic (GSE224218). Data are presented as a statistic (p-value). *p<0.05; **p<0.01; ***p<0.001.

Dataset	PC	Age	Sex	Batch
Dataset 1				
(GSE42861)				
Raw	PC1	−0.016 (0.681)	−1.42 (0.156)	47.8 (<0.001)***
	PC2	0.172 (<0.001)***	1.90 (0.058)	11.0 (<0.001)***
	PC3	−0.261 (<0.001)***	0.242 (0.809)	6.82 (<0.001)***
ComBat	PC1	−0.003 (0.947)	0.905 (0.366)	0.354 (>0.999)
	PC2	0.362 (<0.001)***	−0.558 (0.577)	1.23 (0.107)
	PC3	−0.005 (0.898)	0.056 (0.956)	0.157 (1.000)
iComBat	PC1	0.032 (0.408)	−0.840 (0.401)	0.358 (>0.999)
	PC2	−0.192 (<0.001)***	−2.18 (0.030)*	2.92 (<0.001)***
	PC3	0.057 (0.132)	−5.759 (<0.001)***	0.814 (0.860)
Dataset 2				
(GSE224218)				
Raw	PC1	−0.030 (0.705)	0.590 (0.556)	−3.60 (<0.001)***
	PC2	−0.181 (0.021)*	−0.344 (0.731)	−15.1 (<0.001)***
	PC3	−0.241 (0.002)**	−2.79 (0.006)**	−2.79 (0.010)*
ComBat	PC1	0.010 (0.898)	1.00 (0.319)	−0.015 (0.988)
	PC2	−0.033 (0.674)	1.33 (0.185)	−0.974 (0.336)
	PC3	−0.233 (0.003)**	−2.11 (0.037)*	−0.882 (0.386)
iComBat	PC1	0.011 (0.886)	0.972 (0.333)	0.067 (0.947)
(EPIC → 450 K)	PC2	−0.030 (0.700)	1.349 (0.179)	−0.539 (0.593)
	PC3	−0.224 (0.004)**	−2.09 (0.038)*	−0.702 (0.488)
iComBat	PC1	0.021 (0.794)	0.971 (0.333)	0.527 (0.602)
(450 K → EPIC)	PC2	−0.027 (0.737)	1.420 (0.158)	0.084 (0.933)
	PC3	−0.218 (0.005)**	−1.98 (0.050)*	−0.339 (0.737)
Dataset 3				
(GSE286313)				
Raw	PC1	0.181 (0.129)	−126 (<0.001)***	5.84 (0.001)**
	PC2	−0.750 (<0.001)***	0.200 (0.842)	671 (<0.001)***
	PC3	0.054 (0.652)	0.159 (0.874)	6.54 (<0.001)***
ComBat	PC1	0.191 (0.108)	−185 (<0.001)***	5.82 (0.001)**
	PC2	−0.806 (<0.001)***	0.037 (0.970)	337 (<0.001)***
	PC3	<0.001 (0.999)	0.013 (0.990)	0.566 (0.640)
iComBat	PC1	0.181 (0.129)	−4.39 (<0.001)***	6.51 (<0.001)***
	PC2	−0.776 (<0.001)***	1.71 (0.093)	87.4 (<0.001)***
	PC3	0.208 (0.079)	2.44 (0.019)*	1.35 (0.267)

Table 7 presents the number of differentially methylated CpG sites detected at the Bonferroni-corrected significance level. While fewer CpGs were detected after iComBat correction compared to ComBat (41,677 vs 94,852 for rheumatoid arthritis; 17 vs 127 for smoking history), almost all the CpGs identified after iComBat were also detected after ComBat (40,454 for rheumatoid arthritis; 17 for smoking history).

**Table 7:** Number of differentially methylated CpG sites detected by different batch correction methods.

Dataset	Outcome	ComBat	iComBat	Common
Dataset 1	Rheumatoid arthritis	94,852	41,677	40,454
(GSE42816)	smoking history	127	17	17
Dataset 2	Progression (EPIC → 450 K)	16	13	13
(GSE224218)	Progression (450 K → EPIC)	16	15	14
	Death (EPIC →450 K)	29	24	23
	Death (450 K →EPIC)	29	23	23
	Tumor location (EPIC → 450 K)	4310	4112	4067
	Tumor location (450 K → EPIC)	4310	4182	4093

#### Dataset 2: GSE224218 – intracranial ependymoma EWAS

Fig. 2b shows PCA visualizations for each correction scenario. The raw, uncorrected data exhibited platform-based clustering along both PC1 and PC2. Both ComBat and the two iComBat approaches successfully harmonized these clusters. Figs. 3b and 3c display the sample-to-sample density plots for ComBat versus iComBat (EPIC → 450 K) and ComBat versus iComBat (450 K → EPIC), respectively. Both correlations were over 0.98. As shown in Table 5, GC λ and nSV remained almost unchanged across correction methods. From Table 6 the association of batch with PCs observed in raw data (p<0.001 for PC1 and PC2) was reduced by both ComBat and iComBat, while maintaining the biological associations with age and sex in PC3.

Table 7 shows the differentially methylated CpGs. Similar to Dataset 1, slightly fewer CpGs were detected after iComBat correction compared to ComBat across all outcomes, and almost all the CpGs detected after iComBat were also detected after ComBat. This pattern was consistent regardless of whether 450 K was added to EPIC or vice versa.

#### Dataset 3: GSE286313 – evaluation of epigenetic age

Fig. 2c shows the PCA plots for each correction scenario. Notably, PC1 appears to capture sex-related variation, which remains even after the removal of sex chromosome probes during quality control. Fig. 3d shows the sample-to-sample density plot between ComBat and iComBat corrected data, with a correlation of 0.993. Table 5 shows that both ComBat and iComBat reduced the nSV from 64 in raw data to 1 and 2, respectively. Table 6 shows that while the association of batch with PCs was substantially reduced, the sex-related variation in PC1 and age-related variation in PC2 were retained across all correction methods.

**Fig. 4:**
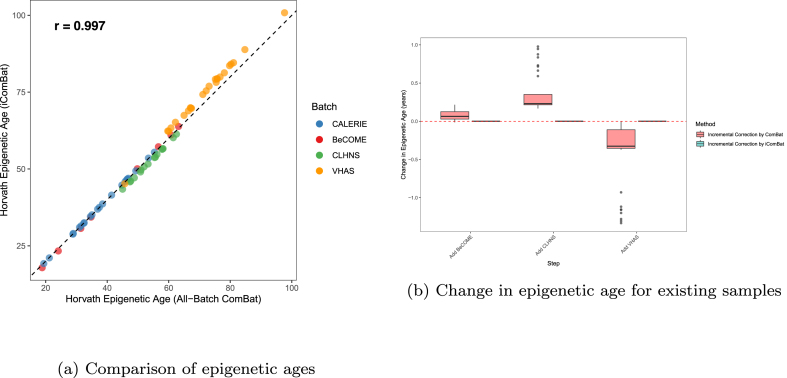
Comparison of epigenetic age between different batch effect correction scenarios in GSE286313 dataset. (a) Scatter plot comparing Horvath epigenetic ages calculated from data corrected by all-batch ComBat versus iComBat. (b) Box plots showing the change in epigenetic age for samples in existing batches when new batches are added incrementally.

Fig. 4a compares epigenetic ages calculated from data after correction of all four batches simultaneously using ComBat versus incremental iComBat correction, showing a correlation of 0.997. Fig. 4b illustrates the change in epigenetic age for samples in existing batches as new batches were added incrementally. With standard ComBat, adding BeCOME resulted in a mean change of 0.077 years with the standard deviation (SD) of 0.60 and the maximum change (MAX) of 2.20, adding CLHNS caused a mean change of 0.36 years (SD=0.26, MAX=0.98), and adding VHAS led to a mean change of −0.39 years (SD=0.38, MAX=1.33). In contrast, iComBat showed zero change in epigenetic age for existing samples when new batches were added.

## 5 Discussion

In this study, we extended ComBat, a widely used method for batch-effect correction of DNA methylation array data, by developing an incremental framework for batch effect correction [20]. The proposed method allows for the correction of new data without reprocessing existing, corrected data. Our numerical evaluations demonstrated that iComBat can achieve batch correction on new data without modifying existing batches, while preserving the power to detect systematic variations associated with treatment. The illustration using actual datasets showed that our method works well on real data.

Our simulation studies demonstrated that iComBat achieved performance nearly equivalent to standard ComBat across various scenarios. Both methods effectively reduced batch effects while preserving case/control signals, and iComBat successfully corrected new batches without modifying existing batch corrections. Notably, we observed that strong covariate effects led to performance degradation in both ComBat and iComBat, particularly in scenario S10 where the covariate effect was extremely strong. This result may suggest that when covariate effects largely exceed biological signals of interest, both methods may have limitations in completely removing these effects while preserving the signals of interest.

The application of iComBat to three actual datasets provided further evidence of its utility. In the EWAS analyses (Datasets 1 and 2), while iComBat detected fewer differentially methylated CpGs compared to ComBat, almost all CpGs identified by iComBat were also detected by ComBat. This indicates that iComBat may maintain high specificity. For epigenetic clock evaluation (Dataset 3), iComBat demonstrated an important advantage. Data corrected by iComBat resulted in epigenetic age estimates that are nearly identical to the data corrected by all-batch ComBat (correlation: 0.997) while maintaining no change in epigenetic age for existing samples when new batches were added. We observed varying patterns across datasets regarding nSV. In Datasets 1 and 2, nSV increased after correction. However, this may reflect that removing batch effects allows previously masked biological variation to become more apparent. The particularly high nSV in Dataset 2 (153–156) may suggest unknown biological variations in this tumor dataset, which is reasonable given the heterogeneous nature of cancer samples from multiple cohorts.

Since ComBat and iComBat are based on linear regression models assuming normally distributed error terms, we recommend using M-values rather than beta-values. The bounded nature of beta-values (bounded between 0 and 1) may violate these assumptions. M-values may better satisfy the normality assumption and provide more appropriate input for the correction algorithms. Both ComBat and iComBat can be applied to data from arrays with Type I and Type II probes, as the analysis is performed on beta-values or M-values rather than raw probe intensities.

The proposed method is expected to be valuable for longitudinal studies involving repeated sample collection and measurements. For example, consider a clinical trial of an anti-aging intervention in which a change in the epigenetic clock is the primary endpoint [32]. When the baseline epigenetic clock value is incorporated into the inclusion/exclusion criteria or used for stratified randomization, it is necessary to evaluate the epigenetic clock values from baseline methylation data. To evaluate the change in the score, the epigenetic clock value was calculated from the methylation data at the end of the trial. However, if a batch effect correction scheme that differs from the one applied at the baseline is used, the measured change may be biased. By employing iComBat, the baseline correction results can be preserved, while consistently correcting data at the end of the trial. This may lead to a more accurate evaluation of the intervention effects.

Some variations in ComBat may be extended to a similar incremental framework. For instance, methods have been proposed in the ComBat framework, which assume that data follow a negative binomial or beta distribution [22], [23]. Their incremental versions may be formulated by following similar principles. Additionally, iComBat can be combined with other batch-effect correction methods. Comparative studies using large-scale longitudinal data have reported that a combination of quantile normalization and ComBat is effective in removing batch effects [33]. A similar preprocessing strategy combined with iComBat may yield more stable results.

## CRediT authorship contribution statement

**Yui Tomo:** Writing – review & editing, Writing – original draft, Visualization, Validation, Supervision, Software, Resources, Project administration, Methodology, Investigation, Formal analysis, Data curation, Conceptualization. **Ryo Nakaki:** Writing – review & editing, Conceptualization.

## Declaration of competing interests

The authors declare the following financial interests/personal relationships which may be considered potential competing interests:

YT served as a technical advisor in statistical science for Rhelixa Inc., from April 2021 to March 2024. RN is the founder and chief executive officer of the company.

## Data Availability

The raw DNA methylation data used in this study are publicly available from the Gene Expression Omnibus under accession GSE42861, GSE224218, and GSE286313.
